# An overview of the electrocardiographic monitoring devices in sports cardiology: Between present and future

**DOI:** 10.1002/clc.24073

**Published:** 2023-06-22

**Authors:** Annachiara Pingitore, Mariangela Peruzzi, Sofia Calaciura Clarich, Zefferino Palamà, Luigi Sciarra, Elena Cavarretta

**Affiliations:** ^1^ Department of General and Specialistic Surgery “Paride Stefanini” Sapienza University of Rome Rome Italy; ^2^ Department of Clinical Internal, Anesthesiology and Cardiovascular Sciences Sapienza University of Rome Rome Italy; ^3^ Mediterranea Cardiocentro Naples Italy; ^4^ Department of Physiology and Pharmacology Sapienza University of Rome Rome Italy; ^5^ Electrophysiology Service, Division of Cardiology Casa di Cura Villa Verde Taranto Italy; ^6^ Department of Clinical Medicine, Public Health, Life and Environmental Sciences University of L'Aquila Coppito Italy; ^7^ Department of Medical‐Surgical Sciences and Biotechnologies Sapienza University of Rome Latina Italy

**Keywords:** arrhythmias, athletes, cardiovascular diseases, ECG monitoring, palpitations, sports cardiology, wearables

## Abstract

**Background:**

Athletes represent a mainly healthy population, which however could be considered at risk of major arrhythmic events, especially in case of undiagnosed cardiomyopathies. For this reason, the periodical sports medicine examination and the electrocardiography are essential tools in the cardiovascular screening, even though they do not always succeed in identifying rhythm disturbances, particularly when asymptomatic or rarely symptomatic.

**Hypothesis:**

Prolonged cardiac monitoring often enables clinicians to stratify the arrhythmic risk and reach the diagnosis. The technological progress of the last decades has produced an always‐increasing number of heart rhythm monitoring devices, starting from the 24‐hour electrocardiogram Holter monitoring and ending with the wide world of wearable devices.

**Methods:**

In the literature, the extreme utility of this equipment in the patients affected by cardiovascular diseases and in the general population is well established. On the contrary, athletes‐based randomized trials or large‐scale epidemiological studies targeting the frequency of cardiac symptoms and the use of cardiac monitoring are missing, while an ever‐growing number of case series and small observational studies are flourishing in recent years.

**Results:**

The present review showcases the available electrocardiographic monitoring options, principally in the medical setting, listing their characteristics, their indications, their supporting evidence, and their general pros and cons.

**Conclusions:**

The ultimate goal of this review is guiding physicians through the wide variety of heart rhythm monitoring options in the specific subfield of sports cardiology, when an arrhythmia is suspected in an athlete, to tailor the diagnostic process and favor the best diagnostic accuracy.

## Graphical abstract

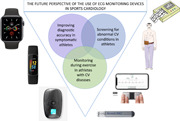

## 1 INTRODUCTION

The intense and continuative exercise training induces multiple physiological cardiovascular (CV) adaptations, better known as the *athlete's heart*. This condition is characterized by several adaptations including an increase in left ventricular (LV) mass; a harmonious remodeling of the cardiac chambers to enhance the cardiac output; the downregulation of sympathetic and upregulation of parasympathetic tone; and the dispersion of ventricular depolarization that is recognizable at the resting electrocardiogram (ECG).^1^

However, aside from these beneficial adaptations, both athletes and sedentary individuals might present early‐stage or covert structural CV diseases that need to be identified to prevent a dramatic event as sudden cardiac death (SCD), which can be triggered by intense exercise and other modulating factors as the competition.^2^ Primary purpose of the preparticipation screening (PPS) is the early identification of cardiac conditions that could potentially cause SCD in athletes. Moreover, it has been demonstrated that physical activity might reveal the arrhythmic substrate and accelerate the progression of some cardiomyopathies, such as the arrhythmogenic cardiomyopathy.^3^ An athlete reporting exercise‐induced symptoms is a rather unusual event, for two different reasons: athletes are generally healthy, but also because they rarely refer symptoms to physicians, even when they are aware of experiencing symptoms, due to the possible disqualification. Hence, when an athlete spontaneously refers palpitations or other symptoms to the team personnel including doctors, suggesting an underlying arrhythmic event, it is imperative to carefully integrate the clinical and instrumental investigations. Nevertheless, some arrhythmic disturbances might also be totally asymptomatic and only identified during the medical examination or SCD might be the first *symptom*.^2^, ^4^ Therefore large‐scale epidemiological studies reporting its prevalence, etiology or prognosis in athletes are missing. Palpitations are frequently referred symptoms in the general population, described as an abnormal perception (irregular, accelerated, intense) of the cardiac pulse; most palpitations occur during sinus rhythm are benign in nature, but also malignant arrhythmias can be the cause of palpitations.^4^ In the literature, the prevalence of palpitations appears to be more common in highly trained or in master athletes, rather than in infants or adolescents.^5^, ^6^ Even if resting palpitations that disappear during exercise is traditionally considered a benign condition, this theory has not been validated in large population‐based prospective studies on athletes. Börjesson et al.^4^ analyzed The SUDDY database (The Swedish study of SUDden cardiac Death in the Young), which comprised 903 cases of SCD in Sweden and reported that 68% of the study population of young individuals (median age 22 years, range 0–35) affected by hypertrophic cardiomyopathy died during recreational or competitive sport, 71% of them experienced possible symptoms before death. On the contrary, other epidemiological studies have demonstrated that in various structural heart disease the worst arrhythmic events happened at rest^7^, ^8^; Finocchiaro et al.^8^ demonstrated that this is particularly true for adolescents, in fact 79% of the 756 cases of SCD in UK adolescents happened at rest and among these, 15% during sleeping time. In addition, some channelopathies, such as long QT type 2 and 3, undermine the threat of SCD particularly during the rest.^7^ Although the presence of palpitations, especially in healthy subjects, is generally associated with a good prognosis, exercise‐induced ventricular arrhythmias should never be neglected, neither when polymorphic, repetitive or with uncommon morphology, since they could represent the first expression of a misdiagnosed cardiomyopathy and potentially trigger a cardiac arrest.^9^, ^10^ Furthermore, the early detection and identification of the palpitations' causes is crucial for guiding the management including the therapeutic strategy, since several complex arrhythmias, such as paroxysmal nodal re‐entrant or tachycardias or secondary to accessory pathways, can be definitively treated with transcatheter ablation.^11^ Palpitations are not the only symptoms eventually referred by athletes, but they can be associated with dizziness, dyspnea or syncope/near syncope. Atrial fibrillation/flutter (AF) are the most common supraventricular arrhythmias in master athletes, male sex and endurance exercise might present additional risks and new‐onset AF can be totally asymptomatic in athletes, especially in master athletes.^6^ They may refer a decrease in performance or in exercise capacity or they can refer the AF identification on a smartphone based‐technology or they can be symptomatic and refer palpitations during effort or at rest.^6^ Although it might be challenging when the events are only occasional, the nature of arrhythmias should always be ascertained by choosing the most appropriate device in the context of an individualized diagnostic process. More specifically, when the PPS, including a stress ECG or even a repeated stress ECG,^10^ is not sufficient in identifying the cause of athletes' symptoms, it is necessary to conduct further investigations that have a greater likelihood of recording the arrhythmic event; the first‐line exam is a 24‐hour ambulatory ECG monitoring even if an occasional symptom might not be recorded. More recently, the vast advent and availability of *wearable* devices and smart‐phone based‐technology have now placed in the hands of the athlete itself the power to diagnose an abnormal change in the heart rate that may hide a malignant arrhythmia. Aim of this review is to evaluate the pro and con of the available technologies for and future perspective for ECG monitoring in the specific setting of sports cardiology.

## 2 TWELVE‐LEAD ECG AND INITIAL CV EVALUATION

A 12‐lead resting ECG is the first step in the evaluation of every athlete or exercise practitioner during PPS and in the evaluation of a symptomatic athlete.^1^ Despite the short duration of the ECG recording (10 seconds) and the difficulties to record a 12‐lead ECG during symptoms, this simple and cost‐effective exam is extremely important to rule out the presence of abnormal findings potentially indicative of a structural cardiac disease, including the suspicious of an accessory pathways, or a channelopathy in an athlete referring symptoms suggestive for an arrhythmic episode. The presence of two or more premature ventricular beats (PVBs) per 10‐second ECG tracing is an abnormal finding in an athlete, subjective to further investigations, similarly to the presence of supraventricular tachycardia, AF, flutter or repetitive ventricular arrhythmias.^1^ In addition, even in the absence of ECG abnormalities, an echocardiographic study can be performed in an athlete with infrequent symptoms to rule out structural heart diseases, possibly related to arrhythmias but not necessarily identified by the sole ECG, as arrhythmogenic mitral valve prolapse.^1^, ^12^

**Figure 1:**
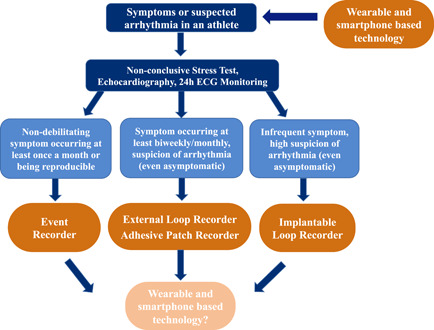
Flowchart of the diagnostic options in an athlete symptomatic for palpitations or other symptoms possibly related to the presence of arrhythmias and their relationship with temporal appearance of the symptoms. The wearables appear on the top of the flowchart (the presence of an underlying arrhythmias is suspected based on the data obtained with the wearable device), but also at the bottom (the use of a wearable device may be useful to monitor and manage the presence of an arrhythmia), but its actual role has not been completely defined.

Moreover, an exercise stress ECG, with systematic blood pressure measurements and fatigue assessment using the Borg scale, is specifically useful to evaluate athletes referring symptoms during efforts or immediately after. In the latter, an abrupt interruption of the test may enhance potential arrhythmias development. If a syncope or a near‐syncope appears immediately after exercise, during the early recovery phase, coupled with hypotension, a reflex mechanism is usually involved, while if it develops during effort, it is a high‐risk feature for a cardiac origin of syncope.^13^ The reproducibility of PVBs (occurrence of the same PVBs morphology and behavior) at a repeated stress ECG is predictive of the presence of a nonischemic LV scar in athletes and should be further evaluated with cardiac magnetic resonance.^10^ Figure 1 presents a diagnostic flowchart in athletes symptomatic for palpitations and Table 1 presents an overview of all available tools.

**Table 1:** Advantages, limitations, and indication of the available ECG monitoring devices.

	Holter ECG	Event Recorders	External Loop Recorders	Adhesive Patch Recorders	Implantable Loop Recorders
Advantages	Low cost Continuous monitoring High resolution and quality of the 12‐lead ECG trace	Recording duration can last for up to 1 month Does not require continuous wear Possible remote cardiac monitoring Well‐tolerated	Recording duration can last for up to 1 month Continuous monitoring Automatic detection of the arrhythmia or activated by the subject Records the onset of the arrhythmia Possible remote cardiac monitoring Well‐tolerated	Recording duration can last for up to 15 days Continuous monitoring Automatic detection of the arrhythmia or activated by the subject Records the onset of the arrhythmia Possible remote cardiac monitoring Very well‐tolerated (waterproof)	Recording duration can last for up to 3 years Does not require the subject's compliance Possible remote cardiac monitoring
Limitations	Brief recording duration (24–48 h) Frequent artifacts due to movement or electrode detachment Possible dermatitis Poorly tolerated for longer monitoring periods Limited storage capacity Limited correlation between symptom and event	Intermediate cost Noncontinuous monitoring Does not record arrhythmias which are asymptomatic/induce disabling symptoms Does not record the onset of the arrhythmia Limited duration of the registration Requires the subject's compliance Single‐lead ECG trace Possible movement artifacts	Intermediate cost Limited data storage Single‐lead ECG trace Possible artifacts due to movement	Intermediate cost Limited data storage Single‐lead ECG trace Possible artifacts due to movement/chest conformation	High costs Minimally invasive surgery for implantation Noncontinuous monitoring Limited data storage
Indications	Initial evaluation of syncope and palpitations occurring on a daily basis or which are reproducible	Palpitations with at least a monthly occurrence, which do not determine disabling symptoms	Palpitations or syncope with at least a monthly occurrence Suspicion of arrhythmic events which could also be asymptomatic Quantification of the arrhythmic burden	Palpitations or syncope with at least a biweekly occurrence Suspicion of arrhythmic events which could also be asymptomatic Quantification of the arrhythmic burden	Infrequent (>1/month) palpitations or syncope Quantification of the arrhythmic burden Search for AF after failure of noninvasive investigation techniques

Abbreviation:  AF, atrial fibrillation/flutter.

## 3 AMBULATORY ECG MONITORING

Short‐term 24‐/48‐hour ambulatory ECG monitoring represents the easiest test that can be performed with a small and lightweight device (about 200–300 g) that allows a 3‐ or 12‐lead continuous noninvasive ECG monitoring. However, symptom frequency is the key in defining the efficacy of this type of diagnostic test, ideally daily or more than weekly episodes. The total number of PVBs or the presence of a high arrhythmic burden is not predictive of a structural heart disease or a malignant arrhythmias, on the contrary athletes with structural heart disease or complex ventricular arrhythmias showed a low arrhythmic burden.^14^ In athletes with PVBs, the use of a 12‐lead ECG monitoring, instead of a 3‐lead, may be less tolerated by the athlete, especially during the effort, but is particularly useful in the diagnosis to detect the PVB morphology (if common or uncommon) and the presence of a high arrhythmic burden (>10% [PVB]/24 hour)^14^; Ensuring that the athlete performs a habitual training of at least 30 minutes during monitoring is of fundamental importance, to determine whether the arrhythmic event is correlated or not with the duration and amount of effort the athlete usually performs.^5^ During pharmacological management and follow‐up even a 3‐lead ECG monitoring can be effective, especially if common or monomorphic PVBs are present. In the occurrence of frequent arrhythmic episodes, short‐term ECG monitoring allows to record the beginning and ending of the arrhythmias, which is particularly useful in the diagnosis and management of supraventricular arrhythmias.^11^ The most common arrhythmia in master athletes is paroxysmal AF and the conditions under that happens, if under high heart rate conditions (i.e., exercise) or under low heart rate conditions (i.e., rest or sleep) holds implication for the choice of the most appropriate medical treatment.^6^ On the contrary, in subjects complaining of dizziness and syncope, short‐term ECG monitoring only has a diagnostic power of only 10% and its use is especially limited in subjects complaining of infrequent palpitations.^13^, ^15^ The repeated and systematic use of the ambulatory ECG monitoring—which is a common practice in the stratification of the arrhythmic risk in patients with structural heart diseases^16^—could partially increase its diagnostic ability in athletes, on the other side, repeated ambulatory 24‐ and 48‐hour ECG monitoring or longer monitoring (up to 7 days) are poorly tolerated by athletes, decreasing compliance, and can record several artifacts during effort. The need to apply chest electrodes constitutes a limitation in long‐term ECG monitoring, which must be taken into account as they can lead to frequent movement artefacts, to potential allergic reactions, and can detach with excessive sweating, as happens during intense physical exercise; furthermore, the use of electrodes is not possible in aquatic sports.

## 4 EVENT RECORDERS

Event recorders consist of a small portable device, which is not worn continuously. When symptoms occur, the recorder is applied directly on the chest or held with both hands and activated by the patient to record a brief, single‐lead ECG trace which is kept in store and, with more modern devices, sent via digital cell phone networks for remote event analysis. Event recorders have proven their cost‐efficacy in patients referring infrequent palpitations in the late '90, compared with conventional 24‐hour ECG Holter monitoring.^17^ The device does not allow storage of a continuous trace and the wearer's participation is essential for the recording, which usually starts after the symptom has begun, so after the onset of the arrhythmia; furthermore, if the subject exhibits an asymptomatic short‐lived arrhythmia or an extremely debilitating symptom, the device would not be activated and therefore would not record. Thus, the main indications for these devices are not debilitating palpitations, with at least a weekly or monthly occurrence or which are inducible in foreseeable conditions (i.e., situational), whereas their use is not indicated in asymptomatic arrhythmias. Limitations of these devices include limited storage capacity of the strips and possible forgetfulness or untimely activation of the recording by the athlete. Battery duration of event recorders varies from a few days up to 1 month.

## 5 EXTERNAL LOOP RECORDER

The external loop recorder is a portable device in which the ECG trace is continuously monitored and updated at regular intervals. There are mainly three activation systems of the device: manual activation in case of symptoms, automatic activation at set intervals, and self‐induced activation in case of predefined rhythms (pauses, bradycardia, supraventricular or ventricular tachycardia, AF, etc.).^18^ In all cases, the device starts recording from before the arrhythmia onset until the end of the arrhythmic event. The recorded data can then be analyzed in real‐time, even from remote. The versatility of this device makes it potentially more effective in identifying both symptomatic and asymptomatic arrhythmias, regardless of their duration, always including the onset of the arrhythmic event itself.^18^ The battery charge of external loop recorders can reach 1 month. In athletes with sporadic symptoms an external loop recorder can be utilized as a second‐line diagnostic investigation for palpitations, because it offers a longer ECG recording, indeed about a 2‐week monitoring is usually sufficient to reach a diagnosis.^11^ The diagnostic efficacy of these devices in the clinical management of athletes symptomatic for palpitations of uncertain origin, occurring at least once a month, has been shown to be 82% with a monitoring duration of only 15 days^11^ and a major arrhythmic event was present in up to 10% of cases. This data is noteworthy if we consider that high‐intensity physical exercise can generate artifacts that compromise the quality of the ECG recording; on the other hand, such encouraging data may also be linked to the inclusion in the study of athletes with relatively frequent symptoms.^11^ However, sensitivity in identifying arrhythmic causes of palpitations could further increase with longer monitoring (e.g., 1 month).

## 6 ADHESIVE CONTINUOUS EXTERNAL MONITORING

Among the new technologies that have entered the external heart rhythm monitoring industry it is noteworthy to mention adhesive continuous external monitoring (Patch‐type Continuous Recorders)^19^ which are positioned on the chest near the left pectoral region for a duration up to 14 days. These devices continuously record 1‐ or 2‐lead ECG traces and can also be activated by the patient, if symptoms are present, for remote monitoring. Several scientific evidence support its use in the general population; these devices have in fact been validated in many clinical studies, which have demonstrated their effectiveness in identifying cardiac arrhythmias and above all episodes of AF.^19^, ^20^ Among all products, the Zio® patch (iRhythm Technologies^©^) had high patient compliance, high analyzable signal time, rates of cardiac arrhythmia detection increased with recording durations >48 h and continued to increase beyond 7 days of recording.^13^, ^20^ The initial experience in athletes is positive, the adhesive devices are comfortable and do not affect the athlete's daily activities, in addition they are water resistant so they can remain in place even during showers and physical exercise.^21^ However, sometimes the adhesive plaster may cause skin irritations, which is the most common adverse reaction of these devices.

## 7 IMPLANTABLE LOOP RECORDERS (ILR)

ILR provide a single lead ECG trace and offer continuous long‐term monitoring. They are small devices that are placed subcutaneously near the left pectoralis major muscle through a minimally invasive surgical procedure under local anesthesia.^22^ The device records in continuous mode and is programmed to automatically archive arrhythmic events with predefined modes (tachycardia, bradycardia, pauses, etc.), as well as to be activated by the symptomatic patients with an external remote control. Therefore, they are designed for recording both symptomatic and asymptomatic events. Furthermore, the ILR can be interrogated remotely, allowing for timely diagnosis and their battery charge lasts up to 3–5 years.^23^

European guidelines recommend the placement of an ILR in many clinical settings, such as syncope or unexplained palpitations after performing noninvasive cardiological tests, as well as for monitoring of individuals at risk of having arrhythmias or in some case of inherited cardiomyopathies,^22^ considering its high diagnostic power (80%–90% in case of palpitations).^23^ ILR is a valid diagnostic tool also for long‐term monitoring in post‐cardiac procedures (i.e., AF ablation) and for patients with noncardiac conditions related to the risk of arrhythmias.^24^ Several studies showed that the use of ILR in patients with stroke provides an additional tool to diagnose AF as the potential cause of cryptogenic stroke, as in patients with severe obstructive sleep apnea with no previous history of AF or in patients with unexplained syncope.^25^, ^26^ The most critical issues related to the use of these devices concern the costs, since IRL is significantly more expensive than external monitoring, and the invasiveness, considering that they might not be well tolerated by young and active subjects such as athletes, despite the market is moving to even smaller devices. However, ILRs show the advantages of being more effective and cost‐effective in reaching a definite diagnosis in patients with unexplained palpitations than conventional ECG monitoring (21% vs. 73%, *p* < .001).^27^ Moreover, ILRs are not damaged by water, so can be placed in athletes engaging in any type of physical activity or training intensity, including water sports,^28^ but the ILR implant may not always be welcomed by the athlete. Nevertheless, even ILRs might show quite an elevated rate of false‐positive events^29^ which varies with the indication to implantation. It should also be noticed that wide population‐based studies on athletes are missing in the literature. Therefore, a case‐by‐case discussion is needed with an accurate selection of the athlete that would benefit the most from an ILR implant and the cost‐efficacy based on the risk of arrhythmias are essential to obtain the ILR best diagnostic power, especially when the use of cheaper devices is possible.

Finally, the duration of the charge allows an almost certain identification of the arrhythmic event even in pauci‐symptomatic subjects. However, since it is a single trace, distinguishing between supraventricular and ventricular arrhythmias may be difficult and oversensing or undersensing phenomena may rashly deplete the memory of the device.^29^

## 8 WEARABLE AND SMARTPHONE‐BASED TECHNOLOGY

**Figure 2:**
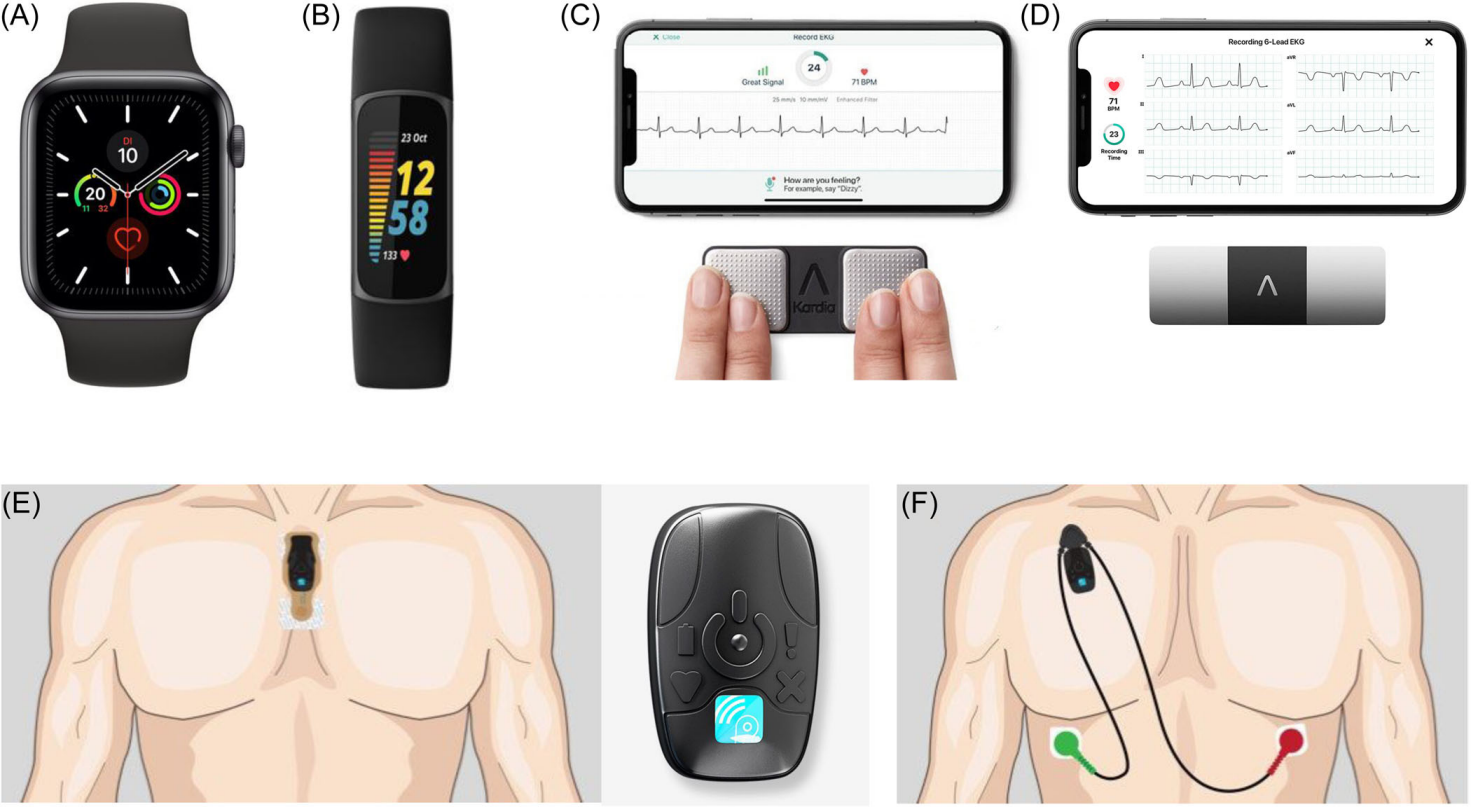
Some examples of commercially available wearable devices: (A) Apple Watch 5; Panel (B) Fitbit charge 5; (C) AliveCorV® KardiaMobile; (D) AliveCorV® KardiaMobile 6L; (E) BodyGuardian™ MINI with strip; (F) BodyGuardian™ MINI PLUS with lead set.

**Figure 3:**
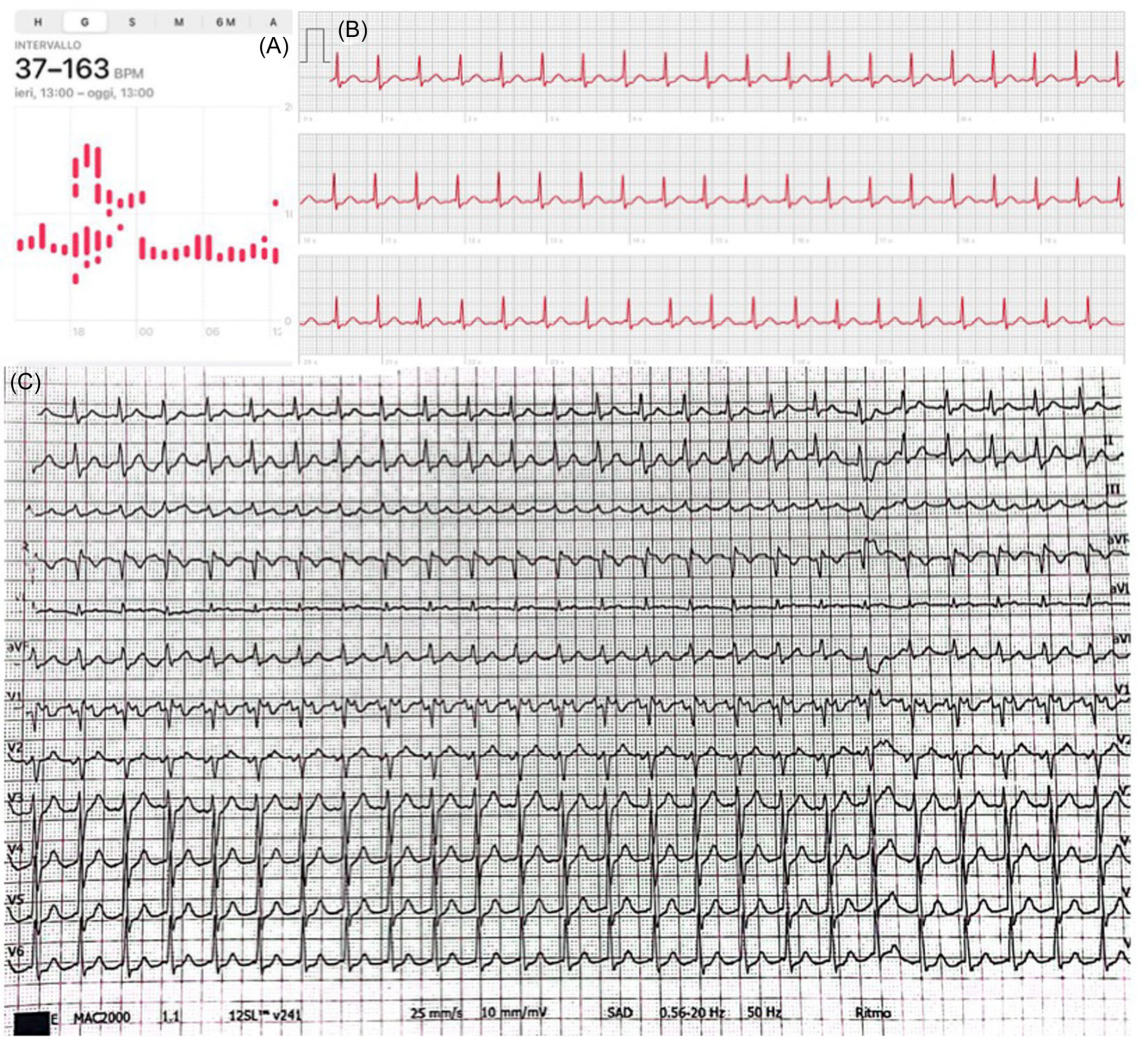
Diagnosis of atrial flutter in an alpine skier. (A) The unexpected rise in the heart rate raise suspicion of an underline arrhythmia. (B) One‐lead electrocardiogram (ECG) tracing demonstrating the presence of atrial flutter. (C) Twelve‐lead ECG obtained after the arrival at the Emergency department that confirmed the diagnosis of atrial flutter. The patient has then been treated accordingly.

In the last decade the technological advancements have allowed a global spread and commercialization of smartwatches and smartphones, with a wide availability of applications and equipment designed to self‐monitor vital parameters, known as *wearable technology* (Figure 2). Indeed, it is so widely used that approximately two‐thirds of the European and North American populations own at least one of these appliances.^30^ Wearables are mainly represented by applications which run directly on mobile phones or on wrist‐worn devices, though sometimes may be integrated in different kinds of leadless accessories, including clothing, smart glasses, smart rings, chest belt, necklaces, headphones, most of them are Food and Drug Administration certified or/and Conformité Européene‐marked.^30^ Currently, the user heart rate and the fitness status are the commonest used applications, albeit usually without healthcare supervision or sanitary approval. The wearables most employed in medical settings operate through photoplethysmography (PPG) or single lead ECG tracings (iECG) and, in the minority of cases, mechanocardiography, which however is less used because unpractical, especially for athletes, and less affordable than other systems.^30^ The PPG is an optical technique that relies on a light source and a photodetector to capture the percentage of nonabsorbed light, which is the expression of the beat‐to‐beat variations. The iECG is composed of two sensors that produce a single‐lead ECG trace (limb or precordial leads, depending on where the sensors are positioned), such as AliveCorV® Kardia (AliveCor®). Recently a new 6‐lead iECG has been released which is equipped of three sensors positioned on both hands and left leg, recording all the limb leads simultaneously (KardiaMobile 6L; AliveCor®); pilot data have showed adequate diagnostic power in identifying rhythm alterations in athletes and T‐waves alternans.^31^ Some devices, such as the latest Apple Watch (Apple Inc) series, rely on both PPG and iECG by holding a finger on the smartwatch digital crown and allowing a 1‐lead ECG and blood pressure monitoring. The diagnostic accuracy of derived data is dependent on the type of the device in use and on the algorithm update, with a sensibility ranging from 88% to 100% and specificity from 81.9% to 99.20%,^32^ provided that physical activity negatively affects its reliability. More specifically, the PPG equipments are more susceptible to artifacts than wireless patches positioned on the chest.^33^ Therefore, wearables can provide a huge amount of data on the heart rate, the heart rhythm and the heart rate variability—whose alterations have proven to be suggestive of arrhythmias and dysfunctions of the autonomic nervous system,^30^ collected during real‐world conditions, across long periods of time and with an adequate reliability, through a continuous or semicontinuous monitoring that often does not require the user activation. Obviously, passive recording devices may be preferred in asymptomatic individuals and vice‐versa, while applications with unlimited storage space would be more appropriate when the symptoms are rare. Some cornerstone studies using the wearable technology focused on the AF screening, including the Apple Heart Study^34^ and the Fitbit Heart Study,^35^ that demonstrated an elevated positive predictive value for detecting AF in subjects receiving irregular rhythm alerts, with the evidence of Apple Watch slightly more performing compared with Fitbit smartwatch (Fitbit International Limited). Of note, even though systematic screening by intermittent ECG is beneficial to detect AF in mature individuals, in the young individuals paroxysmal, asymptomatic AF is a rare event, while it is not infrequent in master athletes, above all in male subjects practicing endurance sports.^6^, ^36^ In Figure 3 we present a case of self‐assessment of a tachyarrhythmia episode in an alpine skier, which was recorded also as 1‐lead ECG and verified with a resting 12‐lead ECG after the access to the Emergency department. Moreover, wearables can be used to seek a wide range of arrhythmias, apart from AF, or ECG trace disorders, as documented by Castelletti et al. who demonstrated an acceptable ability of the BodyGuardian™ MINI Remote Cardiac Monitor (Preventice Solutions Group) in detecting prolonged corrected QT intervals.^37^ Last but not least, multiple‐channel ECGs can be obtained with a commercially available 1‐lead smartwatches, placed in different body positions to obtain nine bipolar ECG tracings (corresponding to Einthoven leads I, II, and III and precordial leads V1–V6).^38^ When the authors compared the diagnostic agreement between the multiple‐channel ECGs obtained by a smartwatch ECG and the traditional 12‐lead ECG, they found a sensitivity of 84% (95% confidence interval [CI], 60%–97%) and specificity of 100% (95% CI, 95%–100%) for the normal ECG tracing; a sensitivity of 93% (95% CI, 82%–99%) and a specificity of 95% (95% CI, 85%–99%) for ST elevation, while for NSTE ECG alterations, sensitivity was 94% (95% CI, 81%–99%) and specificity was 92% (95% CI, 83%–97%).^38^

**Figure 4:**
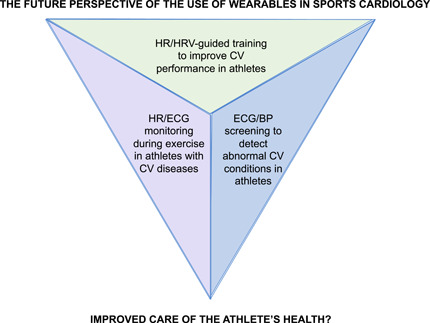
The future perspective in the use of wearables in sports cardiology: the three domains of interest. BP, blood pressure; CV, cardiovascular; HR, heart rate; HRV, heart rate variability.

The most evident advantages of this technology are their wide and cross‐sectional availability, the possibility of a continuous monitoring (24 h/7 days), their small dimensions, costs borne by users only and their resistance to excessive sweating and water exposition. However, their limits exist and are mainly related to the almost complete absence of a remote and systematic control by trained physicians, who in any case should face an enormous burden of data. It must keep in mind that most of wearable are not full‐fledged medical devices and that only the healthcare system equipment should be used for sanitary purposes; moreover, in case of AF, the European guidelines recommend that a physician should confirm the diagnosis by a 12‐lead ECG or a single‐lead trace lasting minimum 30 seconds.^39^ The use of wearables is particularly promising in athletes, as they accept these devices favorably and are used to wear heart rate monitoring or smartwatches, even without medical indication. Engagement of the patient/athlete with the use of digital health technology is particularly advantageous in inducing and maintaining compliance and motivation, even over time, to monitor temporal changes; other key factors for success include usability, perceived utility and value, convenience and health status.^36^, ^40^ The three possible domains pertinent to wearables use are shown in Figure 4 and in Table 2 there is an overview of the different available technologies.

**Table 2:** Mechanisms, use, and limitations of the wearable devices.

	Photoplethysmography sensors (PPG)	ECG sensors	Ballistocardiograms
Mechanism	Detect blood volume changes in the microvasculature	Measure cardiac electrical activity with surface electrodes	Measure body motion generated by the sudden ejection of blood at each cardiac cycle Accelerometers: detect changes in acceleration Gyroscopes: measure angular motions
Use	Adequate for heart rate detection	Gold standard for heart rate detection Gold standard for heart rate variability	Useful for vital sign monitoring
Limitations	Underestimate heart rate in arrhythmias characterized by a lower pulse generation (ie: atrial fibrillation)	Electrode‐skin interface affects signal quality	Accuracy is affected by the site of placement on the human body Accuracy is dependent on the subject's posture (i.e., sleeping or sitting)

Abbreviation: ECG, electrocardiogram.

## 9 FUTURE PERSPECTIVES AND CONCLUSION

The use of wearables has gained momentum not only in everyday life, but also in medicine. In particular, sports cardiology seems the perfect landing for this technology because the integration of continuous assessment of physiological parameters, activity data, and features such as ECG recording can offer an invaluable help in the diagnosis, management, and monitoring of CV diseases. At present, the use of wearable devices can help in raising the clinical suspicion of an arrhythmia or ECG abnormalities; the experiences are still limited but promising. Nevertheless, the clinical identification with a traditional 12‐lead ECG remains the cornerstone in diagnosis and management of arrhythmias or ECG abnormalities. On the other hand, in the next future the use of wearable devices is particularly appealing as monitoring devices to help athletes to personalize training programs, and even to guide exercise prescription, in individuals and athletes with CV diseases. In Figure 2, the three possible domains of interest in sports cardiology are presented, but at present we cannot prove if this approach will contribute improving the CV care of athletes. Evidence of applicability in the three domains are lacking at present, but there is a general excitement in their possible use, so we expect a plethora of studies in the next few years.^41^ Questions raised on the best approach to manage the huge amount of information obtainable with the systematic use of wearables. In this setting, the use of artificial intelligence coupled with machine learning will be the natural evolution, but what will be the physician's role in this new e‐medicine era? Who is going to check, control and manage the data? Moreover, at present most of the wearables entering the market are commercial devices, not health or medical devices. It will be of utmost importance to clearly differentiate those products that are capable to measure ECG and health parameters in a reliable manner, which rely on transparency, validity, and quality of data, to avoid false positive alerts for arrhythmic events that generate anxiety in the athlete and the team physician or further evaluations to rule out arrhythmias, or the worst scenario of an inappropriate disqualification from competitive sport. Only the rigorous research in the amazing field of sports cardiology will answer the question if wearables are of utility or futility.

## CONFLICT OF INTEREST STATEMENT

The authors declare no conflict of interest.

## Data Availability

Data available on request from the authors.
